# Making the case for resistance training in improving vascular function and skeletal muscle capillarization

**DOI:** 10.3389/fphys.2024.1338507

**Published:** 2024-02-09

**Authors:** Mason C. McIntosh, Derick A. Anglin, Austin T. Robinson, Darren T. Beck, Michael D. Roberts

**Affiliations:** ^1^ School of Kinesiology, Auburn University, Auburn, AL, United States; ^2^ Edward Via College of Osteopathic Medicine–Auburn Campus, Auburn, AL, United States

**Keywords:** hypertrophy, skeletal muscle, blood flow, capillaries, angiogenesis, resistance training, brachial artery

## Abstract

Through decades of empirical data, it has become evident that resistance training (RT) can improve strength/power and skeletal muscle hypertrophy. Yet, until recently, vascular outcomes have historically been underemphasized in RT studies, which is underscored by several exercise-related reviews supporting the benefits of endurance training on vascular measures. Several lines of evidence suggest large artery diameter and blood flow velocity increase after a single bout of resistance exercise, and these events are mediated by vasoactive substances released from endothelial cells and myofibers (e.g., nitric oxide). Weeks to months of RT can also improve basal limb blood flow and arterial diameter while lowering blood pressure. Although several older investigations suggested RT reduces skeletal muscle capillary density, this is likely due to most of these studies being cross-sectional in nature. Critically, newer evidence from longitudinal studies contradicts these findings, and a growing body of mechanistic rodent and human data suggest skeletal muscle capillarity is related to mechanical overload-induced skeletal muscle hypertrophy. In this review, we will discuss methods used by our laboratories and others to assess large artery size/function and skeletal muscle capillary characteristics. Next, we will discuss data by our groups and others examining large artery and capillary responses to a single bout of resistance exercise and chronic RT paradigms. Finally, we will discuss RT-induced mechanisms associated with acute and chronic vascular outcomes.

## 1 Introduction

Resistance training (RT) adaptations include increases in skeletal muscle mass, power, and endurance (Deschenes and Kraemer, 2002; Grgic et al., 2022). Neural adaptations occurring during the first few weeks of RT include increased motor unit recruitment and electromyographic activity during maximal contractions (Skarabot et al., 2021). RT promotes increased myofibril protein content and myofiber cross-sectional area in large part due to increases in myofibrillar protein synthesis rates (Roberts et al., 2023).

Beyond increased strength/power and skeletal muscle hypertrophy, evidence suggests RT improves large artery function and skeletal muscle angiogenesis (Zoeller et al., 2009; Beck et al., 2013a; Beck et al., 2013b; Spence et al., 2013; Beck et al., 2014; Verdijk et al., 2016; Holloway et al., 2018b; Naylor et al., 2021; Bovolini et al., 2022). Briefly, the vascular system is characterized by the arterial and venous systems (Pugsley and Tabrizchi, 2000). During ejection of blood from the left ventricle into elastic arteries (i.e., aorta), blood flows from these elastic arteries to a series of large arteries containing a layer of smooth muscle which ensures a rapid distribution of blood to the organ systems (Pugsley and Tabrizchi, 2000). These arteries enter the skeletal muscle bifurcating into smaller arteriolar branches and arterioles. The arterial system transitions to the venous system through arterioles diverging into capillaries. The relationship between RT and vascular adaptations is underappreciated relative to widely examined muscular adaptations. Therefore, the purpose of this review is to examine RT effects on vascular outcomes, vascular assessments, mechanisms underlying RT-induced vascular remodeling, and considerations for future research.

### 1.1 Methods used to assess blood flow and muscle capillarization

**FIGURE 1:**
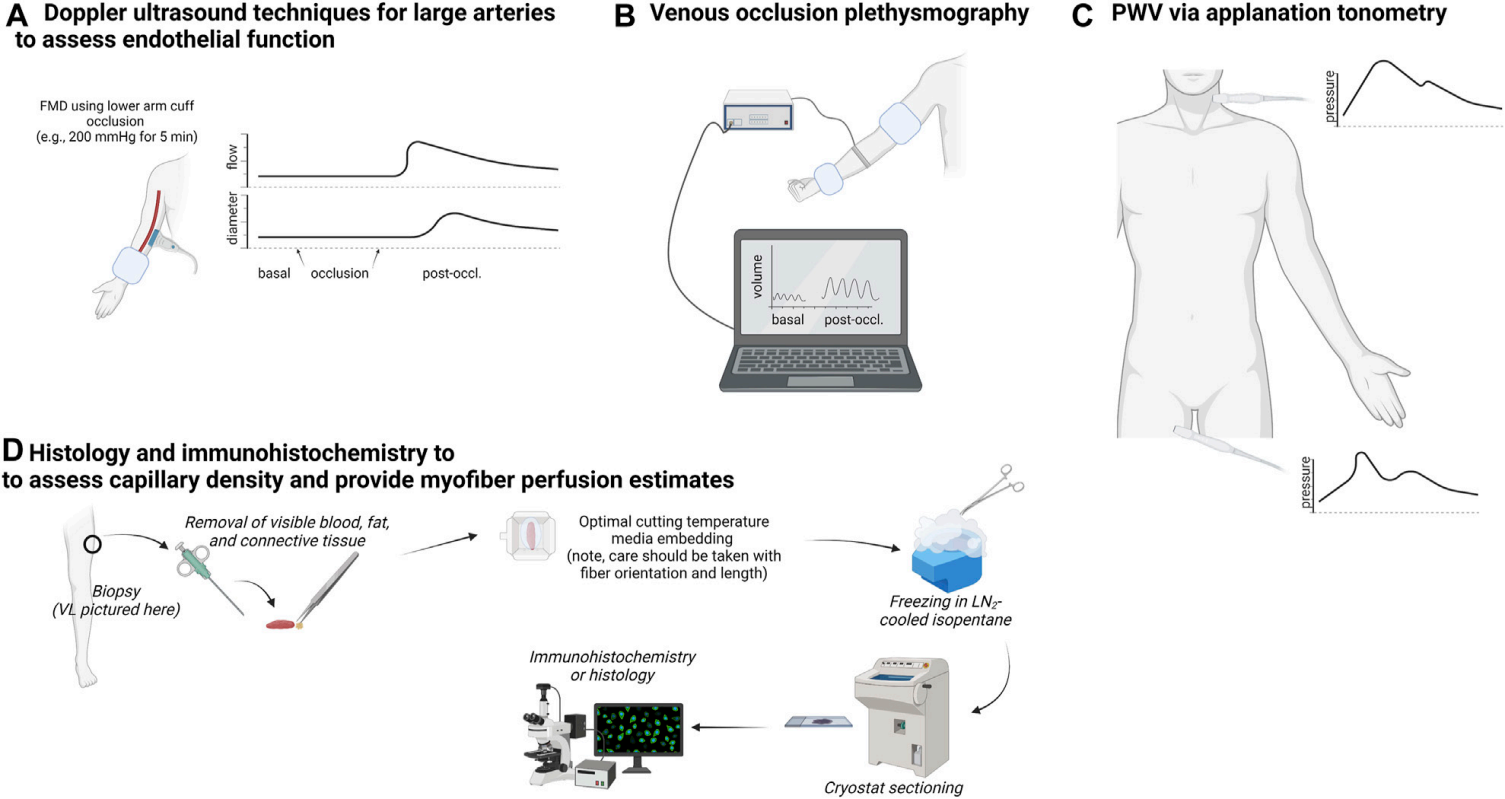
Summary of techniques used to measure arterial function, limb blood flow, and skeletal muscle capillary attributes. Legend: The upper left schematic **(A)** shows techniques that utilize Doppler ultrasound to non-invasively assess large artery blood flow for the extrapolation of endothelial function. The upper center schematic **(B)** illustrates venous occlusion plethysmography, which is used to assess changes in limb blood flow following a series of upper arm and wrist occlusions. The upper right schematic **(C)** illustrates multi-site applanation tonometry to extapolate pulse wave velocity (PWV) to assess arterial stiffness. Finally, the bottom schematic **(D)** shows how a muscle biopsy can be used to assess capillary number per myofiber and extrapolate perfusion indices.

This section provides overviews of laboratory methods to assess vascular function and quantification of capillary characteristics in biopsied muscle to familiarize the reader with techniques discussed in subsequent sections. Figure 1 summarizes these techniques.

### 1.2 Common non-invasive vascular measures

The most widely used method for assessing larger artery blood flow dynamics is flow-mediated dilation (FMD) (Limberg et al., 2020). Briefly, FMD consists of measuring the end-diastolic diameter of conduit arteries and blood velocity using high-resolution ultrasonography at rest and after hyperemic shear stress on the endothelium. Reactive hyperemia is produced after releasing an occlusion cuff inflated for 5 min at ∼200 mmHg below the imaging site. Baseline and hyperemic diameters are compared and increases in lumen diameter are generally attributed to local nitric oxide (NO) release after increased endothelial shear stress. For detailed FMD information, please refer to these reviews (Wray et al., 2013; Limberg et al., 2020).

Regional pulse wave velocity (PWV) is a widely applied and accepted ‘gold standard’ for non-invasive measurement of central and peripheral vessel compliance. PWV is assessed noninvasively by measuring the pulse pressure waveform generated by left ventricular ejection via applanation tonometry and the time delay between two sites (e.g., carotid, and femoral) gated by electrocardiogram (Miyachi et al., 2004; Shirwany and Zou, 2010; Gurovich and Braith, 2011; Mancia et al., 2013; Pereira et al., 2015; Chirinos et al., 2019).

To assess limb blood flow, in the forearm or calf, venous occlusion plethysmography (VOP) is employed (Wilkinson and Webb, 2001). Briefly, VOP involves a mercury in silastic strain-gauge placed around a participant’s limb (e.g., forearm or calf). Limb blood flow is measured at rest and after hyperemia. As the volume within the limb increases, the pulsatile limb circumference is compared against resting values. These estimations are a non-invasive surrogate of microvascular function.

### 1.3 Microscopic assessments for capillary quantification

Capillaries in skeletal muscle deliver nutrients and remove waste products and metabolites (Poole et al., 2013; Olfert et al., 2016). Capillaries are quantified using histology and/or immunohistochemistry, and outcomes include capillary-to-fiber ratio, capillary density, and capillary-to-fiber perimeter exchange index (Olfert et al., 2016; Kissane and Egginton, 2019). The capillary-to-fiber ratio is the ratio of capillaries to myofibers, whereas capillary density represents the number of capillaries within that field of view. The capillary-to-fiber perimeter exchange index is the quotient of the individual capillary-to-fiber ratio and the fiber perimeter (Hepple, 1997). Together, these metrics represent alterations in the capacity for oxygen flux and alterations in any carrier- or receptor-mediated aspect of blood-tissue exchange between the capillaries and myofibers (i.e., nutrient perfusion).

Further, amylase-periodic acid Schiff staining, and histochemical and immunohistochemical staining techniques are performed to assess skeletal muscle capillary number (Qu et al., 1997). CD31 (PECAM-1), a cell adhesion protein highly expressed in vascular endothelial cells, is employed to quantify capillary number in skeletal muscle (Kissane and Egginton, 2019). Together, imaging techniques and quantification of slow and fast twitch myofiber and/or type I and II myosin heavy chain antibodies (Ingjer, 1979; Mitchell et al., 2018) complete typical capillarity analysis.

## 2 Vascular adaptations

### 2.1 Acute resistance exercise effects on conduit artery blood flow

A single RT bout transiently increases heart rate, blood pressure, systemic total peripheral resistance, and blood flow in the large arteries (Miles et al., 1987; Dawson et al., 2013). Blood flow is increased to active skeletal muscle due to reductions in local peripheral resistance termed functional sympatholysis (Rowell, 1997; Thomas and Segal, 2004). Vasoconstrictor responses are reduced in exercising muscle while, as a contradictory reflex, vasoconstriction is increased in resting muscle (Thomas and Segal, 2004). Doppler ultrasonography studies illustrate that femoral artery blood flow transiently increases (i.e., active hyperemia) ∼2-3-fold immediately following an acute bout of RT (Shoemaker et al., 1994; Radegran, 1997). Several lines of independent evidence support loaded muscular contractions transiently increase large artery blood flow and diameter. In our laboratory, immediate post-exercise femoral artery blood flow increases similarly (∼2-fold) in response to lower-load/higher-repetition and higher-load/lower-repetition bouts of leg extensor exercise, and coincides with increases in post-exercise femoral artery diameter (Martin et al., 2017). In a separate study, we observed arm curl RT increases immediate post-exercise brachial artery blood flow 3-fold and brachial artery diameter (∼15%), both subsiding after 15 min of recovery (Fox et al., 2020). While evidence from our group suggests changes rapidly return to pre-exercise levels, recent data suggests increased femoral artery blood flow (∼20%), conductance (∼24%), and diameter (∼5%) can persist up to an hour following knee extensor exercise (Lin et al., 2022).

FMD is considered a primary marker of vascular health and impairment, reduced function, or dysfunction of the endothelium, as measured by FMD, is associated with increased risk of cardiovascular disease and future cardiovascular events (Elliott et al., 1987). Currently, data are mixed, as research groups report brachial FMD (bFMD) is augmented (Gonzales et al., 2011; Franklin et al., 2014; Buchanan et al., 2017; de Oliveira et al., 2020), impaired (Franklin et al., 2014; Choi et al., 2016; Morishima et al., 2018; de Oliveira et al., 2020; Morishima et al., 2020), or unaffected (Jurva et al., 2006; Casey et al., 2007a; Phillips et al., 2011; Buchanan et al., 2017). Discordant reports can be attributed to participant differences in baseline diameter and training status (trained participants being less likely to exhibit a transient reduction sometimes referred to ‘athlete’s artery’ (Green et al., 2012; Zhong et al., 2018). Chronically, RT improves exercise capacity, attenuates the blood pressure response to the increasing workloads, and improves cardiovascular function during graded exercise testing (Lovell et al., 2009). Further, RT reduces central blood pressure and improves microvascular function (Heffernan et al., 2009). Indeed, arterial adaptation to high-pressure loads associated with RT are different and distinctly affect endothelial function when compared with endurance training (Green et al., 2004; Rakobowchuk et al., 2005). It is widely accepted that exercise training augments NO dependent vasodilation of large and small vessels, at least in part, through an upregulation of eNOS protein expression and phosphorylation with the greatest effect occurring during prolonged repetitive endurance exercise (Green et al., 2004). Despite the disparate findings observed in healthy habitual resistance trainers, benefits of RT are widely accepted in prehypertensives, hypertensives, and those at risk for CVD (Beck et al., 2013a; MacDonald et al., 2016; Ogbutor et al., 2019; Pedralli et al., 2020). Further research is imperative to define proper application of exercise modality, length, volume, and intensity targeting populations likely to benefit most while considering baseline FMD status of participants which influence investigation outcomes (Zhong et al., 2018).

### 2.2 Chronic resistance training effects on large artery function

Chronic RT can decrease blood pressure and improve basal blood flow through large arteries (Kelley and Kelley, 2000; Anton et al., 2006; Fragala et al., 2019). Numerous reviews conclude that chronic RT reduces systolic and diastolic blood pressure in healthy and hypertensive younger and older populations (Kelley and Kelley, 2000; Pescatello et al., 2004; Fragala et al., 2019). Indeed, 13 weeks of RT has been reported to increase basal femoral blood flow (∼60%) and vascular conductance in healthy, middle-aged men and women despite no change in the diameter of the lumen (Anton et al., 2006). A study in which over 100 normotensive younger (20–34 years) and middle-aged (36–65 years) men categorized as sedentary or resistance-trained indicate that resistance-trained younger men possess ∼30% higher basal whole leg blood flow compared to untrained counterparts (Miyachi et al., 2005). When comparing inactive controls to highly competitive runners, powerlifters, and weightlifters, weightlifters possessed larger resting brachial arterial diameters (Naylor et al., 2021). Thus, while weeks of RT may not affect large vessel remodeling as indicated above, months-to-years of RT may promote remodeling to increase the diameter of the brachial arteries (and presumably other arteries in trained lower limbs).

Some longitudinal studies suggest RT improves endothelial function in large arteries. In a meta-analysis, which included 51 studies and 2,260 total participants, RT improved endothelial function, and reported a positive correlation with the number of RT sessions and FMD responsiveness (Ashor et al., 2015). Hence, the collective evidence suggests chronic RT can favorably affect blood pressure and vascular function, potentially promoting large artery remodeling which increases vessel diameter.

Arterial Compliance (C) is the change in arterial blood volume (ΔV) due to a change in arterial blood pressure (ΔP) or (C = ΔV/ΔP) (SPENCER and DENISON, 1963). Arterial stiffness is the inverse of arterial compliance. In a compliant vascular system, left ventricle ejection gives rise to lower systolic pressure for a given stroke volume, decreased ventricular wall stress, and reduced myocardial oxygen demand. Arterial stiffness is a major contributing factor for development of cardiovascular diseases with aging, including myocardial infarction and heart failure (Laurent et al., 2001). However, these age-related increases in arterial stiffness are absent or attenuated in regularly exercising adults (Nosaka et al., 2003).

Currently, the beneficial effects of endurance training on arterial compliance in normotensives and hypertensives across all age groups is widely accepted. However, the effects of RT appear to be differential and dependent on training intensity, volume, hypertension status, presence of arterial stiffness, lower limb *versus* upper limb resistance training, age, and health. Specifically, although moderate and low-intensity RT report no unfavorable effects, high-intensity RT has been demonstrated to increase large artery stiffening (Arroyo et al., 1992; Lin et al., 2017). In contrast, others suggest that low and high-intensity RT improve compliance (Casey et al., 2007b; Miura et al., 2008; Okamoto et al., 2011; Beck et al., 2013b; Greenwood et al., 2015; Au et al., 2017).

A 2013 meta-analysis examining RT and arterial stiffness identified 5 studies including 115 young adults and an original article reporting increases in arterial stiffness suggesting potentially unfavorable effects of RT on cardiovascular function (Miyachi, 2013). A 2020 systematic review of 16 studies from 1999 to 2019 and a 2020 meta-analysis and systematic review of 10 studies with 310 total participants reported RT does not alter arterial stiffness in healthy participants (Ceciliato et al., 2020; Garcia-Mateo et al., 2020). Additional studies have reported RT reduces arterial stiffness in young adults (Casey et al., 2007b; Miura et al., 2008; Okamoto et al., 2011; Beck et al., 2013b; Greenwood et al., 2015; Au et al., 2017; Figueroa et al., 2019). Comparisons of outcomes are difficult due to the low number of clinical trials employing RT, differences in age, sex, and current health status of participants and type of RT and measures of compliance. Hence, further investigation is required to resolve these contradictions.

### 2.3 Chronic resistance training effects on skeletal muscle capillarization

Skeletal muscle capillaries are critical for tissue perfusion and delivery of oxygen, nutrients, and removal of waste products (Betz et al., 2021). RT can increase skeletal muscle capillarization, albeit early research in this area in the 1980s did not provide supporting evidence in this regard. For instance, a 1988 review (Tesch, 1988) summarizing studies examining non-exercised controls to weightlifters concluded that “…capillary density decreases consequent to heavy resistance training”, and “…when pronounced hypertrophy of individual muscle fibers occurs, capillary density decreases”. A seminal 8-week longitudinal study by Campos et al. (2002) similarly indicated that neither lower volume, moderate volume, nor higher volume RT affects skeletal muscle capillarization. In contrast, several studies have indicated that 7–12 weeks of RT promotes increases in skeletal muscle capillarization in younger and older participants (McCall et al., 1996; Green et al., 1999; Hostler et al., 2001; Jensen et al., 2004; Verdijk et al., 2016; Nederveen et al., 2017; Holloway et al., 2018a; Holloway et al., 2018b). Interestingly, individuals with less capillaries may display impairments in RT adaptations. For instance, older men with a higher skeletal muscle fiber capillarization prior to 24 weeks of RT experience greater increases in type II skeletal muscle fiber hypertrophy after RT compared to those with lower capillarization (Snijders et al., 2017). Further, increases in type II myofiber satellite cell content following an acute RT bout in the trained state correlate with the degree of type II myofiber capillarization following 12 weeks of prior RT in older participants (Snijders et al., 2019). Hence, the current evidence seemingly suggests that chronic RT increases skeletal muscle capillarization, and this adaptation may optimize skeletal muscle hypertrophy.

## 3 Mechanisms

In consideration of support for RT promoting positive larger artery adaptations and angiogenesis in skeletal muscle, the intent of the subsequent section is to consider potential mechanisms underlying these effects.

### 3.1 Vasodilation mediation

Vasodilation is the widening of the lumen within blood vessels, largely resulting from the relaxation of smooth muscle cells surrounding arterial walls (Kelm, 2002; Egginton and Gerritsen, 2003; Clifford and Hellsten, 2004). Peripheral vessel vasodilation transiently occurs in response to skeletal muscle contractions (Credeur et al., 2015; Hurley et al., 2019), and modulators of this process include NO, prostacyclins, hypoxia, potassium, adenosine, and ATP (Clifford and Hellsten, 2004; Olfert et al., 2016). Further, NO formation is catalyzed by nitric oxide synthase (NOS), derived from L-arginine (Moncada and Higgs, 1993; Clifford and Hellsten, 2004). During skeletal muscle contractions, the increase in shear stress stimulates NO release into circulation and transit to smooth muscle cells from the endothelium and myofibers (Joyner and Dietz, 1997; Clifford and Hellsten, 2004). Moreover, a positive and linear relationship exists between NO production and bFMD in young healthy adults (Casey et al., 2007b). Despite NO being appreciated for its vasodilatory actions, vasodilation is a redundant and complex process with many substances contributing to the balance between vasoconstriction and vasodilation which is outside the scope of this review. The authors direct the reader to an informative thorough review (Clifford and Hellsten, 2004). Continued research is required to parse out how a single bout of RT affects these processes.

### 3.2 Angiogenesis in skeletal muscle in response to resistance training

Vascular endothelial growth factor (VEGF) signaling is an extensively studied mechanism for skeletal muscle angiogenesis. Independent mechanisms induced by RT (e.g., extracellular matrix remodeling through matrix metalloproteases, cytokine signaling, and increases in metabolites) are discussed further herein. Advanced details of VEGF signaling are beyond the scope of the current review, therefore we offer the following for interested readers (Prior et al., 2004; Hoier and Hellsten, 2014; Olfert et al., 2016; Ross et al., 2023). Briefly, VEGF-induced angiogenesis involves VEGF binding to VEGFR2 receptors on endothelial cells which increase proliferation and migration (Ross et al., 2023). Several studies indicate that a bout of RT increases skeletal muscle VEGF mRNA, protein, and plasma protein (Croley et al., 2005; Gavin et al., 2007; Trenerry et al., 2007; Della Gatta et al., 2014). Moreover, current evidence points to myofibers as a prominent site of VEGF production and secretion into the interstitial space and circulation (Hoier and Hellsten, 2014). Multiple factors likely lead to enhanced skeletal muscle VEGF expression in response to a single bout of RT. For instance, transcription factors and transcriptional co-activators including hypoxia inducible factor (HIF)-1α, estrogen-related receptor α (ERRα), peroxisome proliferator-activated receptor gamma coactivator (PGC)-1β and PGC-1α regulate VEGF transcription (Ross et al., 2023). A transcriptomics meta-analysis (Pillon et al., 2020) highlights that each of these genes are upregulated following a bout of RT. Shear stress, induced by RT, has been shown to upregulate myofiber VEGF expression in rodents through NO-mediated mechanisms (Milkiewicz et al., 2001; Baum et al., 2004). Mouse models whereby angiogenesis-related genes are knocked out, knocked down, or deleted (e.g., neuronal NOS or VEGF), demonstrate significant reduction in capillarity and/or skeletal muscle mass (Breen et al., 2008; Baum et al., 2013; Huey et al., 2016; Olfert et al., 2016). Interestingly, RT-induced muscle VEGF expression is lower in older individuals (Croley et al., 2005), and could partially explain age-related impairments in muscle capillarization in response to exercise with aging (Olsen et al., 2020).

Several other notable mediators of angiogenesis exist. For instance, extracellular matrix remodeling through MMPs has been implicated in skeletal muscle capillarization (Ross et al., 2023), and a single bout of RT and chronic RT increase MMP protein expression and/or activity (Wessner et al., 2019; Angleri et al., 2022; Long et al., 2022; Godwin et al., 2023). Tumor necrosis factor-alpha, is a proinflammatory cytokine predominantly produced by monocytes, macrophages, lymphoid progenitor cells, mast cells, endothelial cells, fibroblasts, and neural cells. RT has been shown to acutely increase mRNA expression of TNFα in skeletal muscle (Louis et al., 2007), and may serve a role in angiogenesis by inducing mRNA expression of angiogenic factors, cytokines, proteases, and adhesion molecules (Zubkova et al., 2016). Transforming growth factor-beta signaling, a pathway shown to be induced in skeletal muscle according to transcriptome and DNA methylome analyses in response to RT (Sexton et al., 2023), is also believed to promote endothelial cell differentiation (Lefaucheur et al., 1996). Hence, research will continue to unveil the relevance of these signaling mediators in skeletal muscle capillarization induced by RT.

## 4 Conclusion

While it is well known RT promotes increases in skeletal muscle hypertrophy, strength and power, vascular adaptations and their role in skeletal muscle adaptations to RT is less understood. Perhaps this is due to earlier studies suggesting RT decreases skeletal muscle capillary density and transiently reduces endothelial function. However, emerging evidence suggests RT promotes beneficial vascular adaptations and improves vascular function. Moving forward, it is important to establish whether the vascular adaptations discussed herein are required for optimal RT responses in skeletal muscle and if these relationships hold true across age, sex, and health differences. Nevertheless, this continues to be a fruitful area of discovery.
